# Supplementary material for: Implementation of a marketing plan for the dissemination of the WHO SkinNTDs app in Cameroon
Source: PLoS One. 2025 Sep 25;20(9):e0333295. doi: 10.1371/journal.pone.0333295 (PMC12463274; doi:10.1371/journal.pone.0333295)
Supplement: S1 Appendix — (DOCX) [file pone.0333295.s001.docx]

**Supplementary information file.**

## S1 Appendix. Descriptive of video content and refinement process.

### Narrative draft for video presentations about the WHO SkinNTDs app.

**Script for video A**

**Duration:** 1 minute 19 seconds.

**Language:** French and English.

**Introduction**

Skin diseases are a significant public health problem.

These conditions include neglected tropical diseases, abbreviated as NTDs, which present with skin manifestations. Examples include scabies and leprosy.

These conditions are often an insidious plague on a population due to the lack of early diagnoses that can facilitate fast and effective treatment.

This is why these pathologies are often believed to be mystical illnesses.

WHO has developed a mobile application, the WHO SkinNTDs app, to simplify recognition of the signs and symptoms of these conditions for health workers in all NTD-endemic countries.

**How can I get the app?**

The app is available for free on Google Play and Apple store.

To access it, enter WHO SkinNTDs app in the search bar. The app is easily identifiable as it displays the WHO logo. Then click on install!

**What are its features?**

The app can tell you the disease indicated by a set of signs and symptoms you have identified in a patient. It can then tell you how to treat this pathology.

It will also help you learn more about skin conditions with which you are unfamiliar, and provide treatment advice.

**Script for video B**

**Duration:** 1 minute 08 seconds.

**Language:** French and English.

Skin diseases such as yaws and leprosy are a significant public health problem.

The World Health Organization has developed a mobile application, the WHO SkinNTDs app, to help frontline health workers to easily recognize signs and symptoms of skin-related neglected tropical diseases.

The app also covers other 12 common skin diseases.

The app is available for free on Google Play and Apple store.

To access it, enter WHO SkinNTDs app in the search bar. The app is easily identifiable as it displays the WHO logo.

Once installed, the app is usable offline.

Please note, you must be connected to the internet to change the language.

At app opening, you access the app menu which is also available on top left.

In the ‘Signs and symptoms’ module for example, the app indicates which disease a combination of signs and symptoms corresponds to. It also indicates how to manage the disease.

### Description of the pilot iterative video refinement methodology

1. **VIDEO OPTIMIZATION METHODOLOGY**
2. **Feedback collection process for the video presentations**

- 1. **Initial video evaluation (Input = Video A)**
- Duration: 1 minute 19 seconds.
- Content: Short background on Skin NTDs and the challenges they pose in public health, introduction to the app, installation guide, and app functionalities.
- Format: Voice-narrated (French/English) with static images, background jingle and app screenshots.
- Dissemination: Shared via WhatsApp with 18 purposefully selected participants (13 frontline health workers, 5 academic researchers).
- Diffusion period: Video first released on June 15, 2024.
  1. **Feedback collection approach**
- **Timeline**: Intensive 8-day collection window post-dissemination (June 18-25, 2024)
- **Collection routes** :
  - Structured WhatsApp prompts
  - Spontaneous in-person comments
- **Feedback instrument**: Participants asked to answer 3 questions
  - Is the video clear and understandable?
  - Is the length appropriate?
  - What specific improvements would you suggest?
- **Qualitative analysis**:
  - Thematic coding of all feedback using NVIVO 12 Plus
  - Priority given to recurrent suggestions (>3 mentions)

1. **Phase 2 - Refined video deployment (Output = Video B)**

- Duration: 1 minute 08 seconds.
- Incorporated all Phase 1 feedback.
- Key improvements: Improved video layout including decreasing volume of background jingle and quality of pictures; reduced length by optimizing pace and adapting content straight to the point; added use-case demonstrations; highlighted multilingual feature and offline functionality.
- Deployment: Used in all subsequent dissemination activities without additional formal evaluation.
- Diffusion period: Video first released on July 1, 2024.

1. **FULL RESPONSES COLLECTED**

**Participant 1**

- Not very understandable - requires strong background knowledge to follow
- Introduction needs revision
- Too short
- Too fast
- Enlarge the images and zoom in on lesion areas during explanations

**Participant 2**

1. It's not clear at the beginning, only becomes more understandable as it progresses
2. For me it's okay. The length is normal
3. Slow down the transition speed between images to allow better reading of disease names

**Participant 3**
For me it's clear and understandable. Thank you!

**Participant 4**

- It's a good tutorial and understandable
- I suggest also making tutorials for each menu of the application
- For example, show the steps to follow to make a diagnosis using the app

**Participant 5**
It's OK for me

**Participant 6**
The video is clear and understandable

**Participant 7**

1. Clear and understandable
2. Acceptable but short
3. Improve the quality of dermatological lesion images

**Participant 8**
For me it's very informative. The video is clear

**Participant 9**

1. Clear, understandable and easy to follow
2. Within acceptable time limits

**Participant 10**

1. Clear and understandable, easy to follow
