# Supplementary material for: Implementation of a marketing plan for the dissemination of the WHO SkinNTDs app in Cameroon
Source: PLoS One. 2025 Sep 25;20(9):e0333295. doi: 10.1371/journal.pone.0333295 (PMC12463274; doi:10.1371/journal.pone.0333295)
Supplement: S2 Appendix — (DOCX) [file pone.0333295.s002.docx]

**Supporting information file.**

## S2 Appendix. Definitions of key Google Play Console Analytics metrics.

**Table 1. Definitions of collected variables.**

| **Metrics** | **Definition** |
| --- | --- |
| **User-level metrics** | |
| **Store listing visitors (All users)** | Number of users that visited the store listing and did not have the app installed on any device. Includes new and returning users. |
| **Store listing visitors (New users)** | Number of users that visited the store listing and did not have the app installed on any device. Only includes new users who have never previously installed the app. |
| **Store listing visitors (Returning users)** | Number of users that visited the store listing and did not have the app installed on any device. Only includes returning users who have previously installed and then uninstalled the app from all their devices. |
| **Store listing acquisitions (All users)** | Number of users who visited the store listing and installed the app and did not have the app installed on any device. Includes new and returning users. |
| **Store listing acquisitions (New users)** | Number of users who visited the store listing and installed the app and did not have the app installed on any device. Only includes new users who have never previously installed the app. |
| **Store listing acquisitions (Returning users)** | Number of users who visited the store listing and installed the app and did not have the app installed on any device. Only includes returning users who have previously installed and then uninstalled the app from all their devices. |
| **User acquisition (All users)** | Number of users who installed the app and did not have it installed on any device at that time. Includes users who activated a new device or reactivated a dormant device on which the app was installed. Includes new and returning users. |
| **User acquisition (New users)** | Number of users who installed the app and did not have it installed on any devices at the time. New users who have never previously installed the app. |
| **User acquisition (Returning users)** | Number of users who installed the app and did not have it installed on any devices at the time. Returning users who have previously installed and then uninstalled the app from all their devices. |
| **Installed audience** | Number of users who have the app installed on at least one device that has been turned on in the last 30 days. Includes new and returning users.  Play Console counts unique users with app-installed devices active in the last 30 days, updated daily. |
| **Users loss** | Number of users who have uninstalled the app from all their devices. Includes new and returning users.  "Uninstall" refers to completely removing the app from a device |
| **Daily Active Users (DAU)** | Number of users who opened the app on a given day. |
| **Monthly Active Users (MAU)** | Number of users who opened the app within a 28-day period. |
| **Device-level metrics** | |
| **Device store listing impressions** | Number of unique devices that were used to visit the store listing each day. |
| **Device acquisition (All devices)** | Number of devices on which users installed the app. Includes when a device with the app pre-installed is activated. Includes new and returning devices. |
| **Device acquisition (New devices)** | Number of devices on which users installed the app for the first time. |
| **Device acquisition (Returning devices)** | Number of devices on which users reinstalled the app, having previously installed and then uninstalled. |
| **Install base** | Number of active devices on which the app is installed. An active device is one that has been turned on at least once in the previous 30 days. Includes new and returning devices.  The install base on the last day of the month represents the monthly count (a rolling 30-day window). |
| **Install events** | Number of times the app has been installed, including devices on which the app had previously been installed. Does not include devices with the app pre-installed or device reactivations. Includes new and returning devices. |
| **Daily active devices** | Average number of active devices that open the app each day. An active device is one that has opened the app at least once on any given day. Data is updated daily and averaged over a selected time period (28 days by default). |
| **Device loss** | Number of devices from which the app has been uninstalled. Includes new and returning devices, and when a device becomes inactive. |
| **Uninstall events** | Number of times the app has been uninstalled. Includes new and returning devices, but not when a device becomes inactive. |

**Table 2. Definitions of calculated metrics**

| **Metrics** | **Definition** |
| --- | --- |
| **Store listing conversion rate** | Percentage of users who visit the app store listing and then install the app.  $Store listing conversion rate \left( \% \right)= \frac{Sum of store listing acquisition}{Sum of store listing visitors}x100$ |
| **Device store listing conversion rate** | Percentage of devices that view the store listing and then install the app.  $Device store listing conversion rate \left( \% \right)= \frac{Sum of device acquisition}{Sum of device store listing impressions}x100$  This is a proxy (because of the numerator) as the app can be installed on a device that did not visit the store listing page. For instance, when the app has been installed from an APK obtained elsewhere. |
| **Users to devices ratio** | Ratio of number of devices on which the app is installed per number of users having installed the app.  This ratio indicates the relationship between unique users who acquire the app and the number of devices on which the app is installed. It sheds light on cross-device usage (e.g., if a user installs the app on more than one device).  $Users to devices ratio= \frac{Sum of user acquisitions}{Sum of devices acquisitions}$ |
| **Loss rate** | Ratio of user loss (people who have uninstalled the app from all their devices) to installed audience (people who have the app installed on at least one device that has been turned on in the last 30 days).  This metric helps to assess overall retention at the user level.  $Loss rate \left( \% \right)= \frac{Sum of user loss during the month}{Sum of installed audience on the last day of the month}x100$ |
| **Growth rate** | Ratio that indicates how the user base varies.  $Growth rate \left( \% \right)= \frac{Sum of user acquisition-Sum of user loss during the month}{Sum of installed audience on the first day of the month}x100$ |
| **Net installs** | Captures the number of devices that remain with the app installed.  $Net installs= Sum of install events-Sum of uninstall events$ |
| **Churn rate** | Churn rate (user-level) measures the rate at which users disengage from the app.  $Churn rate \left( \% \right)= \frac{Sum of user loss in the month}{Installed audience on first day of the month}x100$ |
| **User base retention rate** | Measures percentage of users who had the app installed at the beginning of the month and at the end of the month.  This is a proxy for retention as users lost during the month could have been acquired during the month, and thus not counted in the installed audience at the start of the month.  $Retention rate \left( \% \right)=(1- \frac{Sum of user loss of the month}{Installed audience on first day of the month}x100$ |
