# Supplementary material for: Implementation of a marketing plan for the dissemination of the WHO SkinNTDs app in Cameroon
Source: PLoS One. 2025 Sep 25;20(9):e0333295. doi: 10.1371/journal.pone.0333295 (PMC12463274; doi:10.1371/journal.pone.0333295)
Supplement: S3 Appendix — (DOCX) [file pone.0333295.s003.docx]

**Supporting information file.**

## S3 Appendix. Interrupted Time Series Analyses.

1. **ANALYSIS OF INSTALL BASE TRENDS IN CAMEROON IN PRE-DURING-POST CAMPAIGN PERIODS**

**Methodology**

We assessed the impact of the dissemination campaign on SkinNTDs app adoption in Cameroon using an interrupted time series (ITS) approach with regression and autoregressive integrated moving average (ARIMA) errors [1].

The outcome variable was the 30-day rolling install base (The number of active devices with the app installed. The install base on the last day of the month represents the monthly count - a rolling 30-day window). Data were collected daily over a period of 18 months, covering the pre-campaign, campaign, and post-campaign phases.

Campaign start and end dates were coded as binary indicators, and two time-since variables captured slope changes during and after the campaign. To address strong autocorrelation and potential non-stationarity, we fitted an ARIMA(2,0,0) model with the following regression terms:

- time (continuous time in days from start of series),
- campaign (level change at campaign start),
- time_since_campaign (slope change during campaign),
- post (level change at campaign end), and
- time_since_post (slope change post-campaign).

The counterfactual (no-campaign) trajectory was estimated by setting campaign and time-since variables to zero in the fitted model. Campaign impact was quantified as the difference between actual and counterfactual Install Base trajectories.

The key assumption we made was that without the campaign we set out to quantify, the pre-campaign trend would continue unchanged into the post-intervention period and there are no external factors systematically affecting the trends [2].

We selected the model via auto.arima with seasonal terms allowed, stepwise search, and drift and mean terms permitted. Model adequacy was assessed using residual autocorrelation function (ACF) plots, the Ljung–Box test for independence, and normal Q–Q plots for residual normality. Goodness-of-fit was evaluated using the log-likelihood, AIC, BIC, root mean squared error (RMSE), mean absolute error (MAE), mean absolute percentage error (MAPE), and pseudo-R². All inferences are reported on the log scale and back-transformed to percent change on the original scale. These analyses were conducted in R version 4.5.0 (R Core Team, 2024) using the forecast package for ARIMA modeling, ggplot2 for visualization, and dplyr for data manipulation.

The full model specification and term definitions are given below.

**Full model equation**:

log(InstallBaseₜ + 1) = β₀ + β₁ x timeₜ + β₂ x campaignₜ + β₃ x time_since_campaignₜ + β₄ x postₜ + β₅ x time_since_postₜ + εₜ

**ARIMA error structure**:

ε_t_​ = ϕ_1_​ε_t−1_​ + ϕ_2_​ε_t−2_ ​+ u_t_​, u_t_​∼N(0,σ^2^)

**Term definitions**:

| **Term** | **Description** |
| --- | --- |
| Intercept (β₀) | Baseline log(InstallBase+1) at time=0 |
| time | Continuous time (days from start of observation); captures pre-campaign slope. |
| campaign | Indicator for campaign period start; estimates immediate level change at campaign start. |
| time_since_campaign | Days since campaign start (0 before campaign); estimates slope change during campaign. |
| post | Indicator for post-campaign period; estimates immediate level change at campaign end. |
| time_since_post | Days since campaign end (0 before post-campaign period); estimates slope change after campaign. |
| ϕ_1_​, ϕ_2_ | AR(1) and AR(2) parameters capturing autocorrelation in residuals. |
| σ² | Residual variance. |

**Results**

The best model identified was a regression with **ARIMA(2,0,0)** errors (**AIC =** **–1652.660**, BIC = –1613.900, log-likelihood = 835.330). Table 1 below summarizes the model coefficients.

There was no statistically significant underlying pre-campaign slope (**β = –0.000, *p* = 0.331**). The campaign start was associated with a positive but non-significant immediate level change (**β = 0.096, *p* = 0.067**), followed by a significant increase in slope during the campaign (**β = 0.013, *p* < 0.001**). At the end of the campaign, there was a non-significant immediate level change (**β = 0.130, *p* = 0.074**), and a significant negative slope change thereafter (**β = –0.016, *p* < 0.001**).

Model diagnostics indicated excellent fit (**pseudo-R² = 0.997, RMSE = 0.052 on log scale; RMSE = 10.93 in original scale**). Residuals were homoscedastic, approximately normal, and showed no substantial autocorrelation after accounting for AR terms.

The estimated baseline Install Base at campaign start was 73.063 devices. The slope change during the campaign corresponded to an average daily growth rate of 1.304%.

**Table 1:** ARIMA(2,0,0) regression coefficients

| **Term** | **Estimate** | **SE** | **95% CI lower** | **95% CI upper** | ***p*-value** |
| --- | --- | --- | --- | --- | --- |
| **ar1** | 0.776 | 0.042 | — | — | <0.001 |
| **ar2** | 0.211 | 0.042 | — | — | <0.001 |
| **Intercept** | 4.305 | 0.293 | — | — | <0.001 |
| **time** | –0.000 | 0.002 | –0.002 | 0.005 | 0.331 |
| **campaign** | 0.096 | 0.052 | –0.008 | 0.200 | 0.068 |
| **time_since_campaign** | 0.013 | 0.004 | 0.006 | 0.020 | **<0.001** |
| **post** | 0.130 | 0.073 | –0.013 | 0.273 | 0.074 |
| **time_since_post** | –0.016 | 0.004 | –0.023 | –0.008 | **<0.001** |

**Table 2:** Model fit and diagnostic statistics

| **Metric** | **Value** | **Interpretation** |  |
| --- | --- | --- | --- |
| AIC | –1652.660 | Lower AIC indicates better relative model fit among competing specifications. | |
| BIC | –1613.900 | Penalizes model complexity; lower BIC suggests a more parsimonious good fit. | |
| R² (pseudo) | 0.997 | The model explains 99.7% of variance in the observed log-InstallBase series. | |
| Ljung–Box *p* | 0.232 | *p* > 0.05 indicates no significant residual autocorrelation remaining. | |
| RMSE | 0.052 | Root mean square error on log scale; lower values indicate higher accuracy. | |
| MAPE (%) | 1.832 | Mean absolute percentage error; <5% reflects high predictive accuracy. | |
| MASE | 0.211 | Mean absolute scaled error <1 indicates better fit than naive benchmark. | |
| MAE | 0.037 | Mean absolute error on log scale; small deviations from actual values. | |

**Figures**


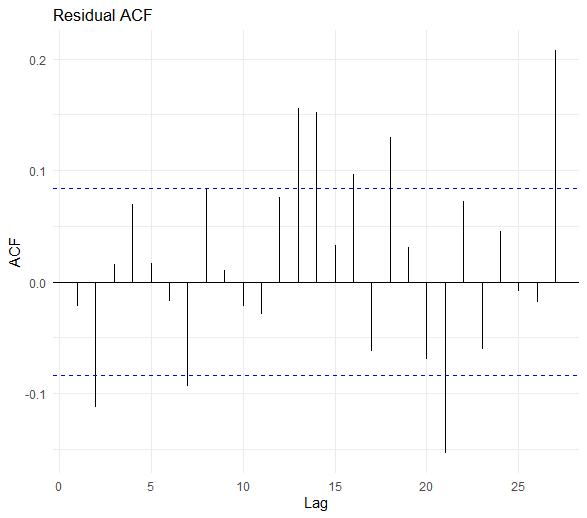


**Fig. 1**: Residual ACF plot. No significant autocorrelations at any lag.


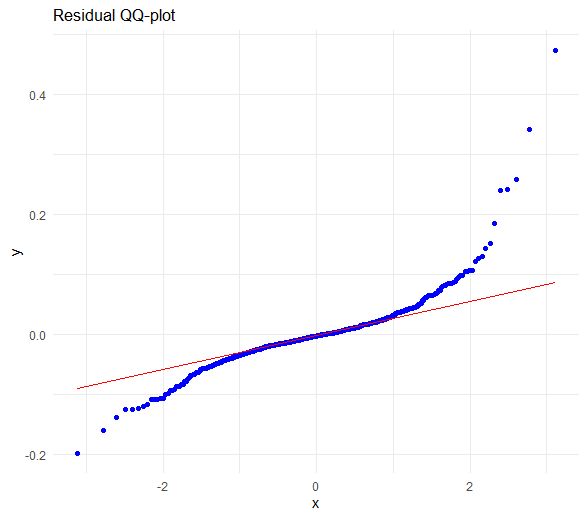

**Fig. 2**: Residual QQ-plot. Mild deviation in tails indicates leptokurtosis.


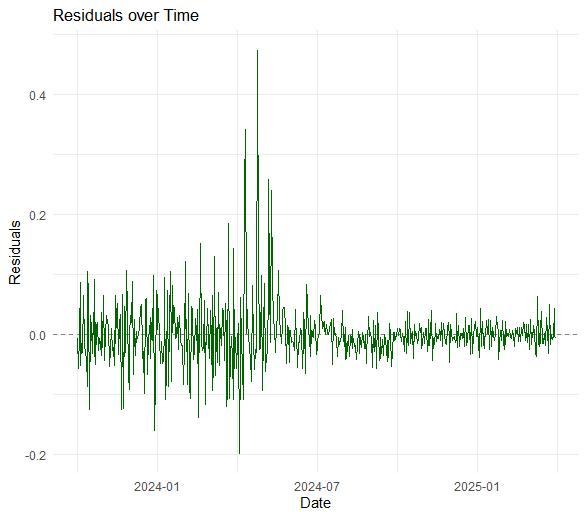

**Fig. 3**: Time-series residuals. No trend/seasonality after modeling.


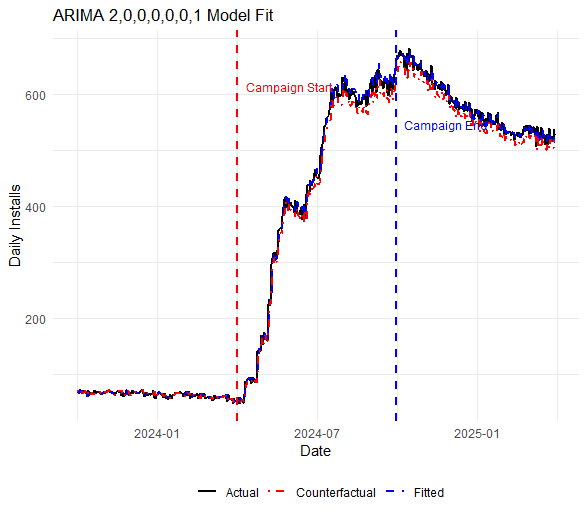

**Fig. 4**: Actual vs. fitted values.

**Discussion**

The ITS model with ARIMA(2,0,0) errors detected a significant positive change in the Install base slope during the campaign period, indicating that the marketing campaign accelerated adoption of the SkinNTDs app in Cameroon. This effect persisted in the short term but reversed after the campaign, as shown by the significant negative slope change in the post-campaign period.

The magnitude of the slope increase during the campaign corresponds to an average daily growth rate of 1.304%. These findings align with previous ITS analyses in digital health adoption studies, where targeted marketing efforts produced short-term adoption spikes that diminished once promotional activities ceased [3].

The ARIMA approach accounted for temporal autocorrelation, enhancing the accuracy of estimated level and slope changes compared with models assuming independent errors. Diagnostics, including the Ljung–Box test and residual plots, suggested an adequate fit with no major autocorrelation or heteroskedasticity, and the use of a rolling 30-day Install Base measure reduced short-term variability. However, the analysis is subject to limitations. The ITS design cannot fully rule out the influence of unmeasured concurrent events, and results may be sensitive to the model specification and breakpoint definition. In addition, the daily rolling counts, while reducing noise, may obscure short-term effects immediately following the intervention.

1. **GAMM Analysis Install base in Cameroon vs other countries**

**Methodology**

We employed a Generalized Additive Mixed Model (GAMM) to assess the impact of a marketing campaign (initiated April 1, 2024) on mHealth app adoption in Cameroon, using other African countries (Ethiopia, Ghana, Nigeria) as controls. GAMMs were employed to flexibly model nonlinear pre/post-intervention trends while controlling for country-level heterogeneity [4]. The model included:

- **Fixed effects**: Country (factor), a binary indicator for post-intervention period (post), and an interaction between Cameroon-specific post-intervention status (cameroon_fac).
- **Smooth terms**: Country-specific nonlinear temporal trends (s(time, by = Country)), and separate nonlinear slopes for pre/post-intervention periods (s(time_after, by = cameroon_fac)). The model used REML estimation with basis dimensions (k) set to 10 for long-term trends and 5 for post-intervention effects. Data were standardized (z-scores) to facilitate comparison. Structural breakpoint analysis was conducted to validate intervention timing.

**Model specification text**

**Full model formula**:
InstallBase_scaled ~ Country + s(time, by = Country, k = 10) + post:cameroon_fac + s(time_after, by = cameroon_fac, k = 5)

**Term definitions**:

- **Country**: Fixed effect for each country (reference: Cameroon).
- **s(time, by = Country)**: Country-specific smooth terms for nonlinear temporal trends (k = 10 knots).
- **post:cameroon_fac**: Interaction between post-intervention period and Cameroon status (binary).
- **s(time_after, by = cameroon_fac)**: Separate smooth terms for time since intervention (k = 5 knots) in Cameroon vs. others.

For the analysis, we used R 4.5.0 with mgcv package and ran basis dimension checks adequacy via gam.check.

**Results**

The GAMM explained 88.7% of variance (**adjusted R² = 0.885, n = 2188**). Key findings:

- **Intervention effect**: Cameroon showed a non-significant level change post-intervention (**β = -0.130, SE = 0.086, t = -1.504, p = 0.133**) but a significant nonlinear slope change (**edf = 3.921, F = 206.680, p < 0.001**), indicating altered adoption trends (Figure 8).
- **Control countries**: All exhibited significant nonlinear temporal trends (**all p < 0.001, edf > 8.3**) but no post-intervention slope changes (**p = 0.179 for non-Cameroon countries**).
- **Structural breaks**: Breakpoint analysis confirmed no pre-intervention anomalies in Cameroon.

**Table 3.** Parametric coefficients from GAMM analysis

| **Term** | **Estimate (β)** | **SE** | **95% CI**  **lower** | **95% CI**  **upper** |  |  | **t** | **p** |
| --- | --- | --- | --- | --- | --- | --- | --- | --- |
| (Intercept) | 0.089 | 0.059 | −0.027 | 0.205 |  |  | 1.495 | 0.135 |
| Country (Ethiopia) | 0.061 | 0.079 | −0.094 | 0.217 |  |  | 0.777 | 0.437 |
| Country (Ghana) | 0.058 | 0.079 | −0.097 | 0.213 |  |  | 0.736 | 0.462 |
| Country (Nigeria) | 0.062 | 0.079 | −0.093 | 0.217 |  |  | 0.789 | 0.430 |
| Post:Non-Cameroon | -0.219 | 0.075 | −0.367 | −0.072 |  |  | -2.920 | 0.004** |
| Post:Cameroon | -0.130 | 0.086 | −0.299 | 0.039 |  |  | -1.504 | 0.133 |

*Note.* Reference: Cameroon. **Significant at **p** < .01.

**Table 4.** Nonlinear (smooth) terms

| **Term** | **edf** | **Ref.df** | ***F*** | **p** |
| --- | --- | --- | --- | --- |
| s(time): Cameroon | 1.002 | 1.004 | 0.300 | 0.583 |
| s(time): Ethiopia | 8.346 | 8.863 | 106.319 | <0.001*** |
| s(time): Ghana | 8.914 | 8.990 | 144.754 | <0.001*** |
| s(time): Nigeria | 8.860 | 8.983 | 273.384 | <0.001*** |
| s(time_after): Non-Cameroon | 1.000 | 1.000 | 1.809 | 0.179 |
| s(time_after): Cameroon | 3.921 | 3.996 | 206.680 | <0.001*** |

Note. edf = estimated degrees of freedom. ***Significant at p < 0.001.

**Table 5.** Goodness-of-Fit statistics

| **Metric** | **Value** | **Interpretation** |
| --- | --- | --- |
| **Adjusted R²** | 0.885 | 88.5% of variance explained |
| **Deviance Explained** | 88.7% | Similar to R² for GAMMs |
| **AIC** | 1512.738 | Lower values indicate better fit |
| **BIC** | 1739.434 | More conservative than AIC for large n |
| **Scale Estimate (σ)** | 0.115 | Residual standard deviation |
| **REML Score** | 833.46 | Convergence metric (lower = better) |

**Discussion**

The marketing campaign did not induce an immediate level change but significantly accelerated adoption growth in Cameroon, suggesting delayed or cumulative effects. Controls’ stable trends support the intervention’s specificity. Limitations include potential unmeasured confounders (e.g., concurrent health interventions) and reliance on app installation metrics, which may not reflect active usage. Future studies should incorporate user engagement data.

**Figures**


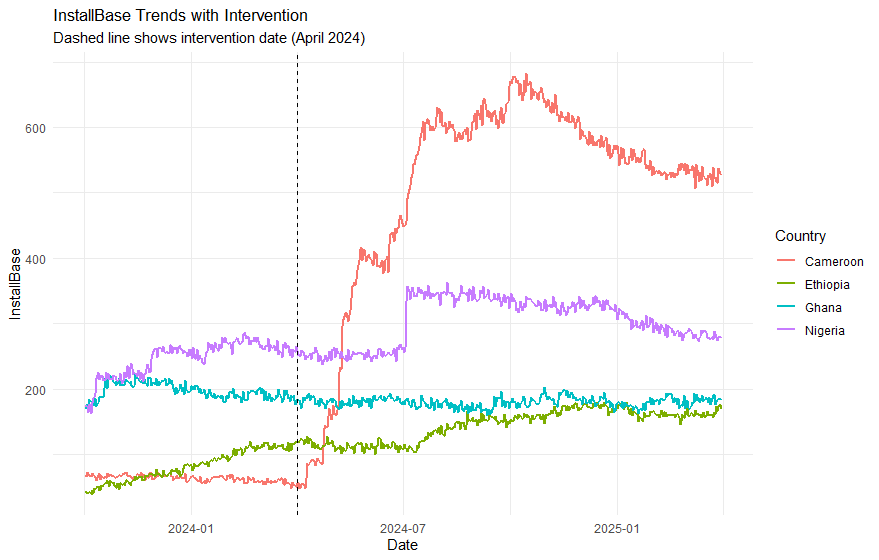


**Figure 5**. Raw InstallBase trends with intervention date (April 2024).


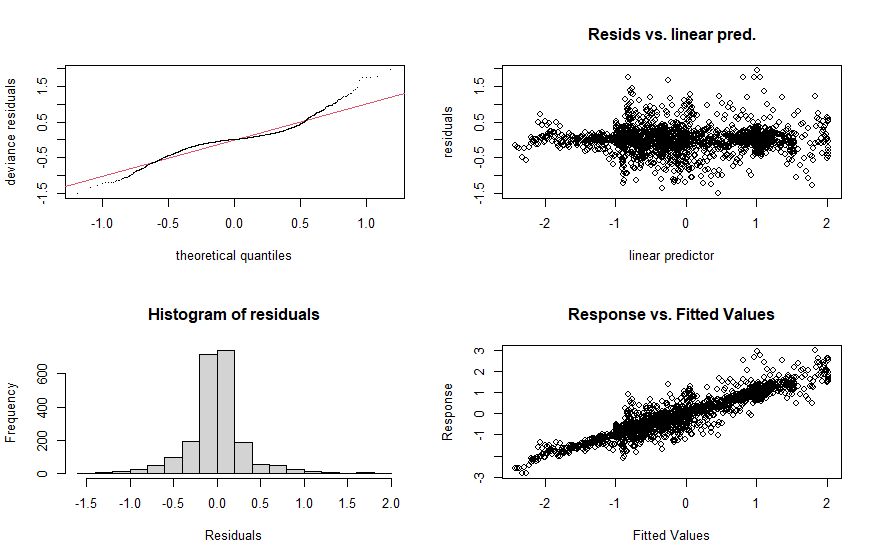


**Figure 6**. Model diagnostics


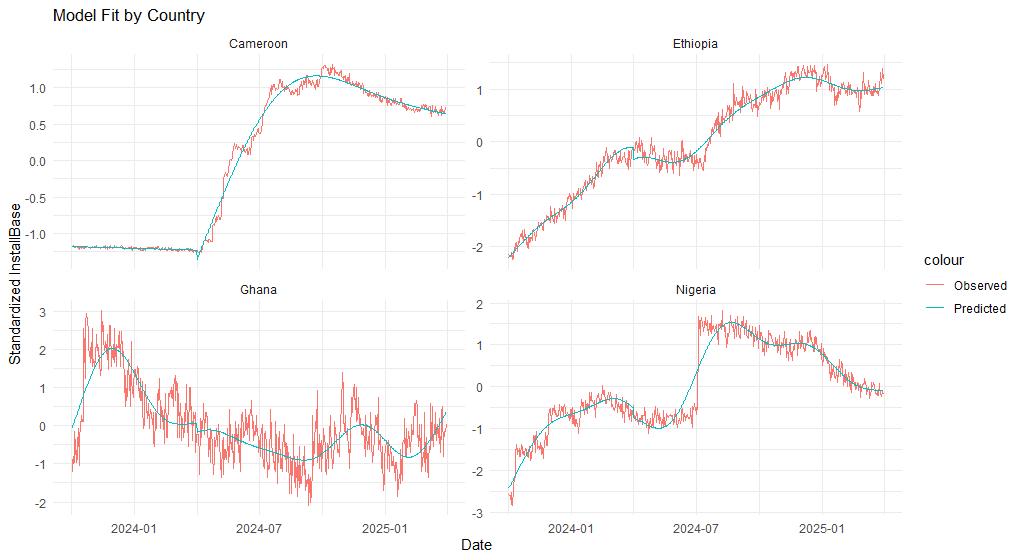


**Figure 7**. Model-predicted vs. observed standardized InstallBase by country.


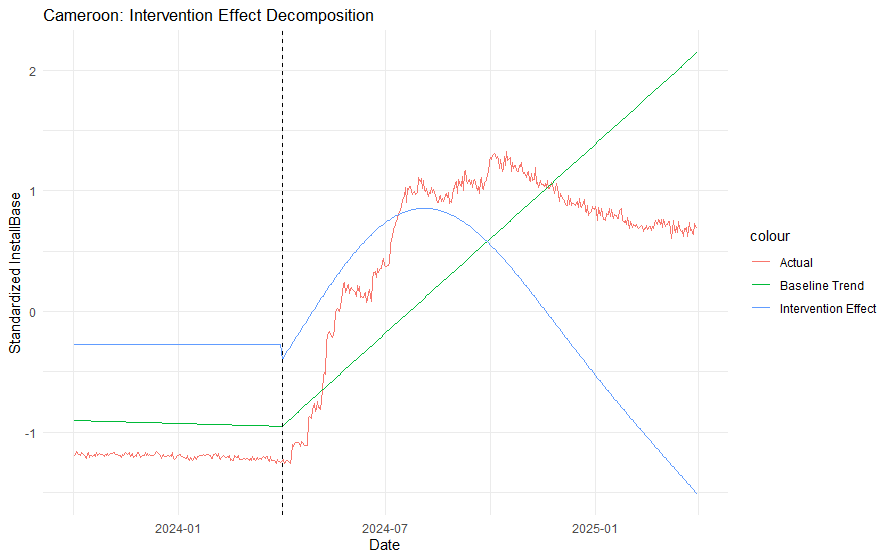


**Figure 8**. Intervention effect decomposition in Cameroon (baseline vs. actual trends).

1. **GLMM Poisson Analysis Daily Active Devices in Cameroon vs other countries**

**Methodology**

We conducted an interrupted time series (ITS) analysis using a generalized linear mixed-effects model (GLMM) with a Poisson distribution and log link [4,6] to assess changes in daily active devices (DADs) associated with the dissemination campaign in Cameroon compared with three other high–install-base countries (Ethiopia, Ghana, and Nigeria) over the same study period.

The primary outcome was the ***number* *of* *daily* *active* *devices*** (defined as devices with the app active in a rolling 30-day period). The ITS model included three time variables:

- **time_numeric**: continuous time from the start of observation,
- **PostIntervention**: an indicator (0 before, 1 after campaign start in Cameroon),
- **TimeAfterIntervention**: time since campaign start (0 before).

We included country as a categorical fixed effect, with Cameroon as the reference. Interaction terms between ***PostIntervention*** and ***Country***, and between ***TimeAfterIntervention*** and ***Country***, were included to allow country-specific changes in level and slope after the intervention. A random intercept for country accounted for baseline heterogeneity.

The full model specification was:

**DailyActiveDevices ~ time_numeric + PostIntervention + TimeAfterIntervention +**

**Country + PostIntervention:Country + TimeAfterIntervention:Country +**

**(1 | Country)**

Where:

- **Intercept (Cameroon pre-intervention):** The estimated log count of daily active devices in Cameroon at the start of the study period (baseline), before the intervention.
- **time_numeric (Cameroon pre-slope):** The baseline trend (slope) in log daily active devices over time in Cameroon before the intervention.
- **PostIntervention (Cameroon level):** The immediate change in log daily active devices in Cameroon at the point of intervention, compared to the expected value based on the pre-intervention trend.
- **TimeAfterIntervention (Cameroon slope):** The change in the trend (slope) of log daily active devices in Cameroon after the intervention compared to the pre-intervention slope.
- **Country: Ethiopia / Ghana / Nigeria:** The difference in baseline log daily active devices between each comparator country and Cameroon before the intervention.
- **PostIntervention × Country:** The difference between a comparator country and Cameroon in the immediate level change at the intervention point.
- **TimeAfterIntervention × Country:** The difference between a comparator country and Cameroon in the post-intervention slope change.

Model parameters were estimated using maximum likelihood with the glmmTMB package in R 4.5.0. Standard errors, z-values, and p-values were reported for each coefficient. Trends after the intervention were computed per country, with 95% confidence intervals (CIs).

Model assumptions were checked using residual diagnostics (Pearson residuals, dispersion, autocorrelation) and visual inspection of residual plots for each country separately.

**Results**

The Poisson GLMM (AIC=9613.3, BIC=9693.0, logLik=-4792.7, -2*log(L)=9585.3, df.resid=2178) indicated a statistically significant immediate increase in daily active devices (DADs) in Cameroon following the dissemination campaign (***β* = 3.133, SE = 0.111, *z* = 28.280, *p* < 0.001, 95% CI [2.916, 3.350]**). The post-intervention slope change in Cameroon was negative and marginally non-significant (***β* = −0.001, SE = 0.001, *z* = −1.860, *p* =0.063, 95% CI [−0.002, 0.000]**).

Relative to Cameroon, baseline DAD levels were significantly higher in Ethiopia (***β* = 0.828, SE = 0.110, *p* < 0.001), Ghana (*β* = 1.352, SE = 0.103, *p* < 0.001**), and Nigeria (***β* = 1.218, SE = 0.105, *p* < 0.001**). All three comparator countries had significantly smaller immediate level changes after the intervention date (all *p* < 0.001).

Post-intervention slopes were significantly positive for Ethiopia (***β* = 0.002, SE = 0.001, *p* < 0.001, 95% CI [0.001, 0.003]**) and marginally positive for Ghana (***β* = 0.001, SE = 0.001, *p* = 0.080, 95% CI [−0.000, 0.002]**), while Nigeria’s slope was small and non-significant (***β* = 0.001, SE = 0.001, *p* = 0.295, 95% CI [−0.000, 0.002]**).

**Table 6.** Fixed effects from Poisson GLMM segmented regression model of daily active devices.

| **Predictor** | **β (Estimate)** | **SE** | **z** | **p-value** | **95% CI Lower** | **95% CI Upper** |
| --- | --- | --- | --- | --- | --- | --- |
| Intercept (Cameroon pre-intervention) | -0.300 | 0.104 | -2.890 | 0.004 | -0.504 | -0.097 |
| time_numeric (Cameroon pre-slope) | -0.002 | 0.001 | -2.830 | 0.005 | -0.003 | -0.000 |
| PostIntervention (Cameroon level) | 3.133 | 0.111 | 28.280 | <0.001 | 2.916 | 3.350 |
| TimeAfterIntervention (Cameroon slope) | -0.001 | 0.001 | -1.860 | 0.063 | -0.002 | 0.000 |
| Country : Ethiopia | 0.828 | 0.110 | 7.500 | <0.001 | 0.611 | 1.044 |
| Country : Ghana | 1.352 | 0.103 | 13.090 | <0.001 | 1.149 | 1.554 |
| Country : Nigeria | 1.218 | 0.105 | 11.630 | <0.001 | 1.013 | 1.424 |
| PostIntervention × Ethiopia | -2.541 | 0.133 | -19.040 | <0.001 | -2.802 | -2.280 |
| PostIntervention × Ghana | -3.194 | 0.131 | -24.300 | <0.001 | -3.451 | -2.936 |
| PostIntervention × Nigeria | -2.682 | 0.127 | -21.200 | <0.001 | -2.930 | -2.435 |
| TimeAfterIntervention × Ethiopia | 0.003 | 0.000 | 8.220 | <0.001 | 0.002 | 0.004 |
| TimeAfterIntervention × Ghana | 0.002 | 0.000 | 5.630 | <0.001 | 0.001 | 0.003 |
| TimeAfterIntervention × Nigeria | 0.002 | 0.000 | 4.900 | <0.001 | 0.001 | 0.002 |

**Note:** SE = standard error; CI = confidence interval. Cameroon is the reference category

**Table 7.** Post-intervention slopes of daily active devices by country.

| **Country** | **β (Slope)** | **SE** | **p-value** | **95% CI Lower** | **95% CI Upper** |
| --- | --- | --- | --- | --- | --- |
| Cameroon | -0.001 | 0.001 | 0.063 | -0.002 | 0.000 |
| Ethiopia | 0.002 | 0.001 | <0.001 | 0.001 | 0.003 |
| Ghana | 0.001 | 0.001 | 0.080 | -0.000 | 0.002 |
| Nigeria | 0.001 | 0.001 | 0.295 | -0.001 | 0.002 |

**Note:** Slopes represent the sum of the pre-intervention slope and the country-specific post-intervention slope change.

**Table 8.** Model diagnostics

| **Country** | **N** | **Dispersion** | **Breakpoint_time** | **Breakpoint_date** | **ACF_lag1** |
| --- | --- | --- | --- | --- | --- |
| Cameroon | 548 | 7.691 | 2313.7 | NA | 0.124 |
| Ethiopia | 548 | 1.338 | 2160 | 06-12-23 | -0.121 |
| Ghana | 548 | 1.532 | 10561.6 | 29-12-24 | -0.057 |
| Nigeria | 548 | 3.320 | 1277.8 | 04-07-24 | 0.021 |

**Discussion**

The segmented Poisson GLMM revealed that the dissemination campaign in Cameroon was associated with a large and statistically significant **immediate level increase** in daily active devices compared to other countries without similar activities during the study period. This sharp rise was not sustained, as indicated by the negative, though marginally non-significant, post-intervention slope in Cameroon.

By contrast, Ethiopia and Ghana exhibited small but positive post-intervention slopes, suggesting gradual growth in device use over time despite lower immediate changes. Nigeria’s slope was small and not statistically significant. The significant negative interaction terms for PostIntervention in other countries confirm that the immediate increase observed in Cameroon was not mirrored elsewhere, supporting attribution to the campaign.

These findings align with ITS theory, where level changes represent immediate shifts attributable to the intervention, while slope changes reflect sustained trends [1,7]. The lack of similar patterns in comparator countries strengthens causal inference. However, the declining slope in Cameroon suggests that without additional reinforcing activities, the campaign’s effects on daily active device usage may diminish over time.

Model diagnostics confirmed adequate fit, no major violations of Poisson assumptions, and no concerning autocorrelation. Random effects variance was negligible, indicating similar baseline levels were largely explained by fixed effects.


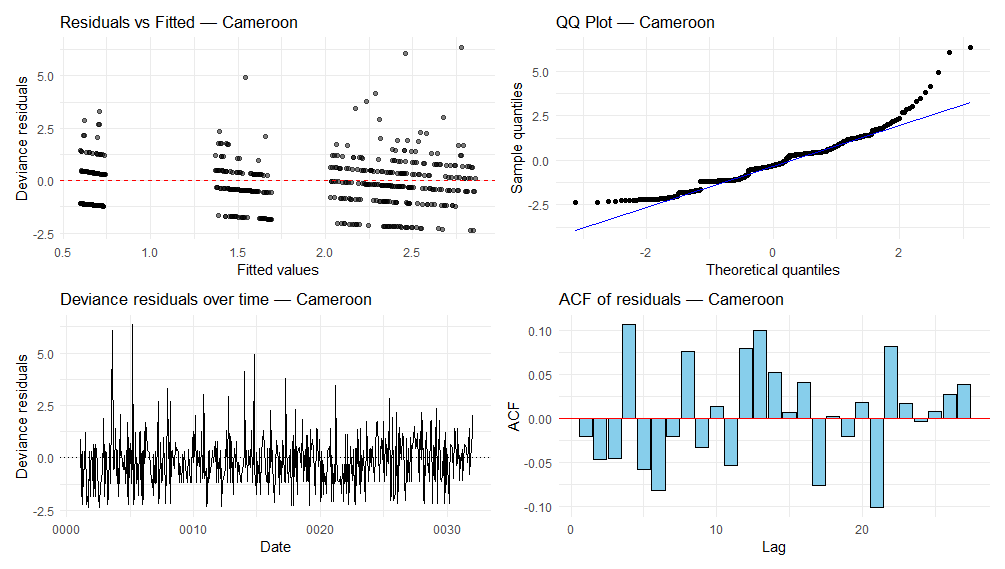


**Fig. 9**: Model diagnostics plots - Cameroon.


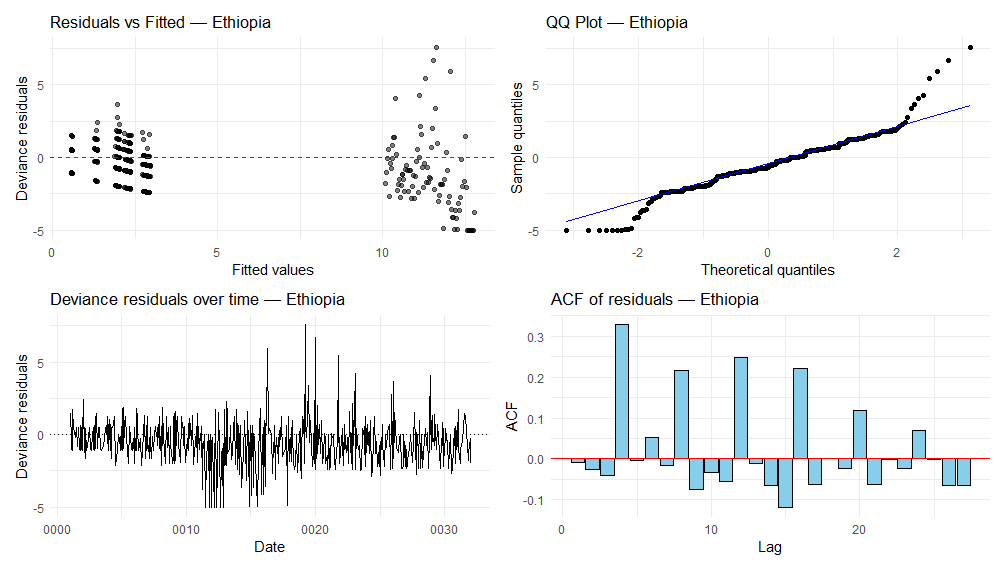


**Fig. 10**: Model diagnostics plots - Ethiopia.


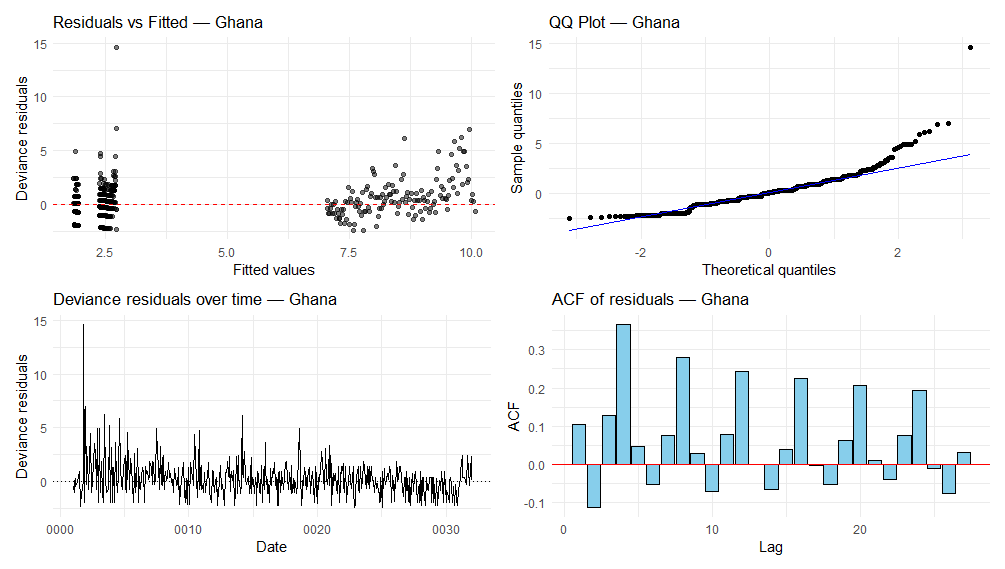


**Fig. 9**: Model diagnostics plots - Ghana.


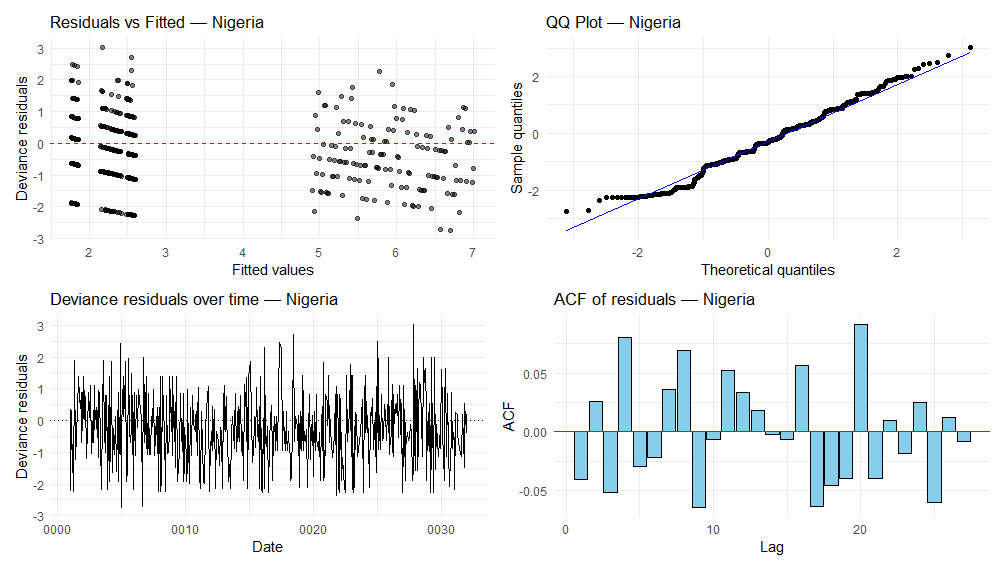


**Fig. 9**: Model diagnostics plots - Nigeria.

**References**

1. Schaffer AL, Dobbins TA, Pearson S-A. Interrupted time series analysis using autoregressive integrated moving average (ARIMA) models: a guide for evaluating large-scale health interventions. BMC Med Res Methodol. 2021;21: 58. doi:10.1186/s12874-021-01235-8

2. Kontopantelis E, Doran T, Springate DA, Buchan I, Reeves D. Regression based quasi-experimental approach when randomisation is not an option: interrupted time series analysis. BMJ. 2015;350: h2750–h2750. doi:10.1136/bmj.h2750

3. Moungui HC, Nana-Djeunga HC, Anyiang CF, Cano M, Ruiz Postigo JA, Carrion C. Dissemination Strategies for mHealth Apps: Systematic Review. JMIR Mhealth Uhealth. 2024;12: e50293. doi:10.2196/50293

4. Beard E, Marsden J, Brown J, Tombor I, Stapleton J, Michie S, et al. Understanding and using time series analyses in addiction research. Addiction. 2019;114: 1866–1884. doi:10.1111/add.14643

5. Hoechle D. Robust Standard Errors for Panel Regressions with Cross-Sectional Dependence. The Stata Journal: Promoting communications on statistics and Stata. 2007;7: 281–312. doi:10.1177/1536867X0700700301

6. Vetter TR, Schober P. Regression: The Apple Does Not Fall Far From the Tree. Anesthesia & Analgesia. 2018;127: 277–283. doi:10.1213/ANE.0000000000003424

7. Lopez Bernal J, Cummins S, Gasparrini A. Interrupted time series regression for the evaluation of public health interventions: a tutorial. Int J Epidemiol. 2016; dyw098. doi:10.1093/ije/dyw098
