# Supplementary material for: Implementation of a marketing plan for the dissemination of the WHO SkinNTDs app in Cameroon
Source: PLoS One. 2025 Sep 25;20(9):e0333295. doi: 10.1371/journal.pone.0333295 (PMC12463274; doi:10.1371/journal.pone.0333295)
Supplement: S4 Appendix — (DOCX) [file pone.0333295.s004.docx]

**Supporting information file.**

## S4 Appendix. Draft of a marketing plan for the WHO SkinNTDs app

## **Objectives**

The marketing strategy will focus on achieving the following objectives:

- **Increase awareness:** Reach ≥80% of target FHWs in North/Far-North Cameroon via WhatsApp, emails, or in-person sessions within 6 months.
- **Training coverage:** Conduct ≥20 in-person sessions.
- **Drive downloads:** Achieve ≥60% conversion rate from store visits to downloads.
- **Sustain engagement:** Maintain ≥80% user retention 3 months post-installation.

## **Orientation questions to prepare the marketing plan**

1. ***What services are you providing via the WHO SkinNTDs app?***

Below are the functionalities provided by the WHO SkinNTDs app.

**Table 1. The functionalities of the WHO SkinNTDs app.**

| **Functionality** | **Description** | **Behavior/action required from users** |
| --- | --- | --- |
| Description of signs and symptoms of skin diseases | Explains the signs and symptoms of skin diseases including sNTDs. Indicates the most likely diagnosis. | Open “**Signs and symptoms**” module and read. |
| Diagnoses | Interactively guides users to learn about case diagnosis based on the information they provide. | Click “**Diagnoses**” module and answer questions. |
| Case management | Indicates how to manage a suspected or confirmed sNTDs case. | Navigate the “**How to manage**” module and read. |
| Glossary | Provides a definition of each key word used in the app. | Click on “**Glossary**” module and read. |
| Skin NTDs Learning Hub | Provides an overview of sNTDs including worldwide distribution, an additional route to sNTDs diagnosis, information about the app, and links to additional resources on the web. | Navigate “**Skin NTDs Learning Hub**”. |

1. ***What use cases are available or envisaged?***

The user can interact with the app at any time, including during a patient consultation. The app provides information about the signs and symptoms presented by a patient, and can run an interactive diagnostic and indicate the most likely diagnosis. Once the user has assessed the pathology of a patient, the app can provide information on how to manage the condition. The user can also navigate through different app modules to learn more about sNTDs in general.

1. ***Who are the target users?***

The target users are FHWs (doctors, nurses, or assistant nurses) who provide healthcare to clients almost every day in areas endemic for NTDs in general, especially sNTDs. As the prevalence of sNTDs may be unknown in some areas, the target users are not restricted to those in areas known to be endemic for sNTDs. These FHWs could be working in urban, semi-urban, or rural areas. Remote semi-urban and rural settings may have limited and unreliable access to electricity, the internet, and dermatological specialists, and be hard to access due to a peripheral location and undeveloped infrastructure.

1. ***What is the most effective way to reach these target users?***

With the rise of mobile phone subscriptions, we make an assumption that most people will have their own smartphone, even in semi-urban and rural areas. Therefore, given the physical inaccessibility of these areas, conducting a marketing campaign for the app through in-person interactions with a central team from the Ministry of Public Health, or a local or international organization, is not recommended. We envisage conducting a muti-channel marketing campaign that will use a mix of communication channels to reach target users, depending on their setting and the resources available.

1. ***What is the most effective message for these users? What matters most to them?***

Use of mHealth apps is relatively new within the general population of clinicians in Cameroon, and initiatives promoting the use of mHealth apps are rare. Thus, this is a relatively virgin marketplace.

To identify the most effective message and discover what matters most to the users we will conduct A/B testing of several versions of the same advertisement, distributed to different groups, with different designs, color coding, calls to action, and message content, to determine which version produces the highest conversion rate.

## **Strengths, Weaknesses, Opportunities, and Threats (SWOT) analysis of the app**

1. ***Strengths of the WHO SkinNTDs app?***

- Quality of graphics and ease of use.
- Multilingual support.
- Reputable source.

1. ***Weaknesses of the WHO SkinNTDs app?***

- Other competing mHealth apps offering similar functionalities, such as the eSkinHealth app (<https://derma.jmir.org/2023/1/e46295>).

1. ***Opportunities for the WHO SkinNTDs app?***

- Renewed global efforts towards sNTDs eradication that could boost support and demand.

1. ***Threats to the WHO SkinNTDs app?***

- Low electricity and internet coverage in the remote areas where most sNTDs cases are located.
- FHWs can uninstall the app from their personal devices at any time.

## **Dissemination activities**

Dissemination will be carried out in three implementation stages: (i) launching the app; (ii) engaging users for continuous app use; (iii) embedding the app in routine clinical practice.

1. ***Launching phase***

Objective: to launch the app on the health market within the targeted population.

Period: January to February 2024.

1. ***Engagement phase***

Objective: to encourage users to continuously use the app.

Period: February to June 2024.

1. ***Embedment phase***

Objective: to foster the integration of the app within routine health system activities.

Period: June 2024.

The table below summarizes the activities that will be implemented during the launch of the app.

**Table 2. List of activities to be implemented during the launching phase.**

| **Communication channel** | **Objective** | **Expected result** | **Targeted people** | **Who should be involved** | **Message** | **Date/period** |
| --- | --- | --- | --- | --- | --- | --- |
| **E-mail** | Announce the launching of the app | People aware that the app will be launched | Government NTDs program managers and officers at central and regional levels and in non-governmental organizations (NGOs) | Head of national NTDs coordination unit, focal point for research at NTDs coordination unit, research team | “***Dear all, we announce a meeting to launch the WHO SkinNTDs app for use in [country]***” | Jan 15, 2024 |
| **One-day in-person / online meeting**  Place: Governmental NTDs coordination unit meeting room or any other hall | Officially launch the app | Central level government staff and NGOs are informed about the existence of the app, the download link, functionalities, and general information about the app (developer, WHO, donor). | Government NTDs program managers and officers at central and regional levels and NGOs working on NTDs in the country | Head of national NTDs coordination unit, focal point for research at NTDs coordination unit, research team | Participants discuss dissemination plan and contribute by validating messages prepared to promote the app | Jan 23, 2024  (1 day) |
| **WhatsApp community & WhatsApp groups** | Keep target users engaged | Target users engaged in the research and app use | Healthcare providers | Research team | Call-to-action (reminders to download the app/use it), technical support | Jan 15 to Jun 30, 2024  (~6 months) |
| **Flyers**  (handed to participants in-person at meetings, conferences, and any other opportunity) | Infographics and key message about the existence of the app, and its main value.  Include a QR code and/or text to indicate download link. | Target users download the app | Potential target base users (healthcare providers and day-to-day users of the app) | Head of national NTDs coordination unit, focal point for research at NTDs coordination unit, research team | “***The SkinNTDs app makes you smarter at recognizing Skin NTDs***” | Jan 15 to Jun 30, 2024  (~6 months) |
| **Print advertising** | Announcement in a specialized health journal in the country (e.g: “***Echos Sante***” journal in Cameroon) | Government stakeholders and target users are informed about the existence of the app, download link, functionalities, general information about the app (developer, brand owner, sponsor). | Government stakeholders and target users | Head of national NTDs coordination unit, focal point for research at NTDs coordination unit, research team | “***The Ministry of Health and WHO introduce the SkinNTDs app, a novel app that makes frontline healthcare providers smarter at recognizing Skin NTDs***” | Jan 30 (for the celebration of international world NTDs day) |
| **Short promotional video spot**  **(duration: 1 to 3 min)** | Video spot on the app, its functionalities and added value | Government stakeholders and target users are informed about the existence of the app, download link, functionalities, general information about the app (developer, brand owner, sponsor). | Government stakeholders and target users | Head of National NTDs coordination unit, focal point for research at NTDs coordination unit, research team | “**The MOH and the WHO introduce the SkinNTDs app, a novel app that makes frontline healthcare providers smarter at recognizing Skin NTDs**” | Jan 30 ( 1 day: for the celebration of international world NTDs day) |
| **Face-to-face interactions: in-person ad-hoc (unplanned) encounters** | Sell the app (highlight its main value) and install it on user devices.  Explain how to use the app and discuss tips. | Target users install the app, are engaged in the research and use of the app | Government stakeholders and target users | Head of National NTDs coordination unit, focal point for research at NTDs coordination unit, research team | Call-to-action (reminders to download the app/use it, tips), technical support | Jan 15 to Jun 30, 2024  (~6 months) |
| **Push notifications** (to be embedded in the app by the developers) | Keep people that have installed the app engaged | People that have installed the app, are using the app (engagement) | Target users (people who have downloaded the app) | Developers  should include this feature in the app (**Push notifications** should be programmed as **just-in-time notifications**: adapted to users’ behaviors and availability) | Just-in-time call-to-action to use the app (in working hours) and reminders with tips (in non-working hours) about identifying sNTDs cases.  For instance, a message could pop up saying “***do you know that the app contains definitions of common dermatological signs?***”. | Jan 15 to Jun 30, 2024  (~6 months) |

***Additional considerations for crafting promotional messages***

- **Use messages that go beyond describing app functionalities**. These messages should focus on the benefits of the app for FHWs and how it could best serve them in routine practice.
- As suggested by prior research, social media engagement typically occurs during non-working hours. Push notifications will be sent to those that have already downloaded the app but only used it a few times or not at all. The message for these notifications will consist of a reminder to use the app and relate to identifying sNTDs cases. For instance, a message could pop up saying “***do you know that the app contains definitions of common dermatological signs?***”.
