# Supplementary material for: Implementation of a marketing plan for the dissemination of the WHO SkinNTDs app in Cameroon
Source: PLoS One. 2025 Sep 25;20(9):e0333295. doi: 10.1371/journal.pone.0333295 (PMC12463274; doi:10.1371/journal.pone.0333295)
Supplement: S5 Appendix — (DOCX) [file pone.0333295.s005.docx]

**Supporting information file.**

## S5 Appendix. Detailed results of statistical tests.

**Purpose of the analysis**

These analyses examine the statistical association between the independent variable *period* (pre-campaign, campaign and post-campaign) and six dependent variables:

1. **Store listing conversion rate**
2. **Device conversion rate**
3. **Growth rate**
4. **Loss rate**
5. **Churn rate**
6. **User base retention rate**

This analysis is to determine whether the campaign has significantly influenced these metrics and how these metrics varied between periods. We averaged growth rate, loss rate, churn rate and user base retention rate for each 6-months period.

1. **Store listing conversion rate: all users vs period**

| **ANOVA - Store listing conversion rate: all users** | | | | | |  |
| --- | --- | --- | --- | --- | --- | --- |
|  | **Sum of Squares** | **df** | **Mean Square** | **F** | **p** | **ƞ^2^** |
| **Period** | 0.188 | 2 | 0.0938 | 0.556 | 0.574 | 0.006 |
| **Residuals** | 33.735 | 200 | 0.1687 |  |  |  |

**Assumption checks**

| **Homogeneity of variances test (Levene's)** | | | |
| --- | --- | --- | --- |
| **F** | **df1** | **df2** | **p** |
| 13.5 | 2 | 200 | <.001 |

| **Normality test (Shapiro-Wilk)** | |
| --- | --- |
| **Statistic** | **p** |
| 0.851 | <.001 |

| **One-way ANOVA (non-parametric): Kruskal-Wallis** | | | | |
| --- | --- | --- | --- | --- |
|  | **χ²** | **df** | **p** | **ε²** |
| **Store listing conversion rate: all users** | 1.75 | 2 | 0.416 | 0.00868 |

**Post Hoc Tests : Period**

| **Period** | **Mean Difference** | **SE** | **df** | **t** | **p_tukey_** | **Cohen's d** | **95% CI Lower** | **95% CI Upper** |
| --- | --- | --- | --- | --- | --- | --- | --- | --- |
| **Pre-campaign vs Campaign** | -0.001 | 0.088 | 200 | -0.016 | 1.000 | -0.003 | -0.428 | 0.421 |
| **Pre-campaign vs Post-campaign** | -0.072 | 0.100 | 200 | -0.725 | 0.749 | -0.176 | -0.655 | 0.303 |
| **Campaign vs Post-campaign** | -0.079 | 0.069 | 200 | -1.026 | 0.561 | -0.172 | -0.504 | 0.159 |

**Note. Comparisons are based on estimated marginal means**

**Interpretation:** normality and homogeneity assumptions violated. Kruskal-Wallis test found no difference in store listing conversion rate across periods confirmed with post-hoc tests.

**Reference table. Interpreting effect size values** *(Source: Maher JM, Markey JC, Ebert-May D. The Other Half of the Story: Effect Size Analysis in Quantitative Research. CBE—Life Sci Educ. 2013;12: 345–351. doi:10.1187/cbe.13-04-0082)*

| **Effect size measure** | **Small**  **effect size** | **Medium**  **effect size** | **Large**  **effect size** | **Very large**  **effect size** |
| --- | --- | --- | --- | --- |
| **Cohen’s d (or one of its variants)** | 0.20 | 0.50 | 0.80 | 1.30 |
| **Pearson’s r** | 0.10 | 0.30 | 0.50 | 0.70 |
| **Eta-squared (ƞ^2^)** | 0.01 | 0.06 | 0.14 |  |
| **Epsilon-squared (ε²)** | 0.01 | 0.06 | 0.14 | >0.26 |

1. **Store listing conversion rate: new users vs period**

| **ANOVA - Store listing conversion rate: New users** | | | | | |  |
| --- | --- | --- | --- | --- | --- | --- |
|  | **Sum of Squares** | **df** | **Mean Square** | **F** | **p** | **ƞ^2^** |
| **Period** | 0.193 | 2 | 0.0966 | 0.567 | 0.568 | 0.006 |
| **Residuals** | 32.716 | 192 | 0.1704 |  |  |  |

**Assumption checks**

| **Homogeneity of variances test (Levene's)** | | | |
| --- | --- | --- | --- |
| **F** | **df1** | **df2** | **p** |
| 10.2 | 2 | 192 | <.001 |

| **Normality test (Shapiro-Wilk)** | |
| --- | --- |
| **Statistic** | **p** |
| 0.857 | <.001 |

| **One-way ANOVA (non-parametric): Kruskal-Wallis** | | | | |
| --- | --- | --- | --- | --- |
|  | **χ²** | **df** | **p** | **ε²** |
| **Store listing conversion rate: new users** | 0.973 | 2 | 0.615 | 0.00501 |

**Post Hoc Tests : Period**

| **Period** | **Mean Difference** | | **SE** | | **df** | | **t** | | **p_tukey_** | | **Cohen's d** | | **95% CI Lower** | | **95% CI Upper** | |  |
| --- | --- | --- | --- | --- | --- | --- | --- | --- | --- | --- | --- | --- | --- | --- | --- | --- | --- |
| **Pre-campaign vs Campaign** | | -0.082 | | 0.094 | | 192 | | -0.879 | | 0.654 | | -0.199 | | -0.647 | | 0.248 | |
| **Pre-campaign vs Post-campaign** | | -0.111 | | 0.105 | | 192 | | -1.057 | | 0.542 | | -0.270 | | -0.774 | | 0.235 | |
| **Campaign vs Post-campaign** | | -0.029 | | 0.071 | | 192 | | -0.410 | | 0.912 | | -0.071 | | -0.411 | | 0.269 | |

**Interpretation:** normality and homogeneity assumptions violated. Kruskal-Wallis test found no difference in Store listing conversion rate for new users across periods confirmed with post-hoc tests.

1. **Store listing conversion rate: returning vs period**

| **ANOVA - Store listing conversion rate: returning users** | | | | | |  |
| --- | --- | --- | --- | --- | --- | --- |
|  | **Sum of Squares** | **df** | **Mean Square** | **F** | **p** | **ƞ^2^** |
| **Period** | 0.174 | 2 | 0.0872 | 1.08 | 0.355 | 0.077 |
| **Residuals** | 2.104 | 26 | 0.0809 |  |  |  |

**Assumption checks**

| **Homogeneity of variances test (Levene's)** | | | |
| --- | --- | --- | --- |
| **F** | **df1** | **df2** | **p** |
| 6.70 | 2 | 26 | 0.004 |

| **Normality test (Shapiro-Wilk)** | |
| --- | --- |
| **Statistic** | **p** |
| 0.617 | <.001 |

| **One-way ANOVA (non-parametric): Kruskal-Wallis** | | | | |
| --- | --- | --- | --- | --- |
|  | **χ²** | **df** | **p** | **ε²** |
| **Store listing conversion rate: returning users** | 2.34 | 2 | 0.310 | 0.0837 |

**Post Hoc Tests : Period**

| **Period** | **Mean Difference** | | **SE** | | **df** | | **t** | | **p_tukey_** | | **Cohen's d** | | **95% CI Lower** | | **95% CI Upper** | |  |
| --- | --- | --- | --- | --- | --- | --- | --- | --- | --- | --- | --- | --- | --- | --- | --- | --- | --- |
| **Pre-campaign vs Campaign** | | 0.163 | | 0.133 | | 26.0 | | 1.22 | | 0.450 | | 0.574 | | -0.403 | | 1.550 | |
| **Pre-campaign vs Post-campaign** | | -5.13e−16 | | 0.184 | | 26.0 | | -2.80e−15 | | 1.000 | | -1.78e−15 | | -1.327 | | 1.327 | |
| **Campaign vs Post-campaign** | | -0.163 | | 0.157 | | 26.0 | | -1.04 | | 0.557 | | -0.574 | | -1.716 | | 0.569 | |

**Interpretation:** normality and homogeneity assumptions violated. Kruskal-Wallis test found no difference in store listing conversion rate for returning users across periods.

1. **Device conversion rate: all users vs period**

| **ANOVA - Device conversion rate: all users** | | | | | |  |
| --- | --- | --- | --- | --- | --- | --- |
|  | **Sum of Squares** | **df** | **Mean Square** | **F** | **p** | **ƞ^2^** |
| **Period** | 3.46 | 2 | 1.728 | 3.73 | **0.026** | 0.035 |
| **Residuals** | 94.57 | 204 | 0.464 |  |  |  |

**Assumption checks**

| **Homogeneity of variances test (Levene's)** | | | |
| --- | --- | --- | --- |
| **F** | **df1** | **df2** | **p** |
| **0.561** | **2** | **204** | **0.571** |

| **Normality test (Shapiro-Wilk)** | |
| --- | --- |
| **Statistic** | **p** |
| **0.908** | **<.001** |

| **One-way ANOVA (non-parametric): Kruskal-Wallis** | | | | |
| --- | --- | --- | --- | --- |
|  | **χ²** | **df** | **p** | **ε²** |
| **Device conversion rate: all users** | 9.28 | 2 | **0.010** | 0.045 |

**Dwass-Steel-Critchlow-Fligner pairwise comparisons (associated with Kruskal-Wallis) : device conversion rate: all users vs period**

|  |  | **W** | **p** |
| --- | --- | --- | --- |
| **Pre-campaign** | **Campaign** | 2.02 | 0.325 |
| **Pre-campaign** | **Post-campaign** | 3.71 | **0.024** |
| **Campaign** | **Post-campaign** | 3.47 | **0.038** |

**Interpretation:** normality assumption violated. Kruskal-Wallis test found a difference in device conversion rate for all users across periods (χ^2^=9.28, df=2, p=0.010, ε^2^=0.045). Dwass-Steel-Critchlow-Fligner pairwise comparisons further **confirmed that difference in device conversion rate (specified as the pre-specified primary outcome) between Pre-campaign vs Post-campaign (p=0.024) and Campaign vs Post-campaign (p=0.038) periods**.

1. **Growth rate: proxy: avg vs period**

| ANOVA - Growth rate: proxy: avg | | | | | |
| --- | --- | --- | --- | --- | --- |
|  | **Sum of Squares** | **df** | **Mean Square** | **F** | **p** |
| **Period** | 7122 | 2 | 3561 | 4.89 | **0.023** |
| **Residuals** | 10929 | 15 | 729 |  |  |

**Assumption checks**

| Homogeneity of variances test (Levene's) | | | |
| --- | --- | --- | --- |
| **F** | **df1** | **df2** | **p** |
| 12.9 | 2 | 15 | <.001 |

| Normality test (Shapiro-Wilk) | |
| --- | --- |
| **Statistic** | **p** |
| 0.809 | 0.002 |

**One-way ANOVA (non-parametric)**

| Kruskal-Wallis | | | | |
| --- | --- | --- | --- | --- |
|  | **χ²** | **df** | **p** | **ε²** |
| **Growth rate :proxy :avg** | 10.0 | 2 | **0.007** | 0.591 |

**Dwass-Steel-Critchlow-Fligner pairwise comparisons**

| Pairwise comparisons - Growth rate: proxy: avg | | | |
| --- | --- | --- | --- |
|  |  | **W** | **p** |
| **Campaign** | **Post-campaign** | -4.08 | **0.011** |
| **Campaign** | **Pre-campaign** | -3.40 | **0.043** |
| **Post-campaign** | **Pre-campaign** | 1.13 | 0.703 |

**Interpretation:** normality assumption violated. Kruskal-Wallis test found a difference in growth rate for all users across periods (χ^2^=10.0, df=2, p=0.007, ε**^2^**=0.591). Dwass-Steel-Critchlow-Fligner pairwise comparisons further **confirmed that difference in growth rate between Campaign vs Post-campaign (p=0.011) and Campaign vs Pre-campaign (p=0.043) periods**.

1. **Loss rate: proxy: avg vs period**

| ANOVA - Loss rate: proxy: avg | | | | | |  |
| --- | --- | --- | --- | --- | --- | --- |
|  | **Sum of Squares** | **df** | **Mean Square** | **F** | **p** | **ƞ^2^** |
| **Period** | 34.2 | 2 | 17.11 | 6.18 | **0.011** | 0.452 |
| **Residuals** | 41.5 | 15 | 2.77 |  |  |  |

**Assumption checks**

| Homogeneity of variances test (Levene's) | | | |
| --- | --- | --- | --- |
| **F** | **df1** | **df2** | **p** |
| 1.70 | 2 | 15 | **0.216** |

| Normality test (Shapiro-Wilk) | |
| --- | --- |
| **Statistic** | **p** |
| 0.967 | **0.735** |

**Post hoc analysis: Periods**

| **Period** | **Mean Difference** | | **SE** | | **df** | | **t** | | **p_tukey_** | | **Cohen's d** | | **95% CI Lower** | | **95% CI Upper** | |  |
| --- | --- | --- | --- | --- | --- | --- | --- | --- | --- | --- | --- | --- | --- | --- | --- | --- | --- |
| **Pre-campaign vs Campaign** | | -0.61 | | 0.96 | | 15.0 | | -0.64 | | 0.80 | | -0.37 | | -1.61 | | 0.87 | |
| **Pre-campaign vs Post-campaign** | | -3.18 | | 0.96 | | 15.0 | | -3.31 | | **0.01** | | -1.91 | | -3.35 | | -0.47 | |
| **Campaign vs Post-campaign** | | -2.57 | | 0.96 | | 15.0 | | -2.68 | | **0.04** | | -1.55 | | -2.92 | | -0.17 | |

**Interpretation:** no assumption violated. ANOVA found a difference in loss rate across periods (p=0.011, ƞ**^2^**=0.452). Post hoc tests comparisons further **confirmed that difference in loss rate between Campaign vs Pre-campaign (p_tukey_=0.012, Cohen’s d=-1.912, very large negative effect size) and Post-campaign vs Pre-campaign (p_tukey_ =0.043, Cohen’s d=-1.546, very large negative effect size) periods**.

1. **Churn rate: proxy: avg vs period**

| ANOVA - Churn rate: proxy: avg | | | | | |  |
| --- | --- | --- | --- | --- | --- | --- |
|  | **Sum of Squares** | **df** | **Mean Square** | **F** | **p** | **ƞ^2^** |
| **Period** | 48.3 | 2 | 24.17 | 8.53 | **0.003** | **0.532** |
| **Residuals** | 42.5 | 15 | 2.84 |  |  |  |

**Assumption checks**

| Homogeneity of variances test (Levene's) | | | |
| --- | --- | --- | --- |
| **F** | **df1** | **df2** | **p** |
| 2.81 | 2 | 15 | **0.09** |

| Normality test (Shapiro-Wilk) | |
| --- | --- |
| **Statistic** | **p** |
| 0.972 | **0.842** |

**Post hoc analysis**

| **Period** | **Mean Difference** | | **SE** | | **df** | | **t** | | **p_tukey_** | | **Cohen's d** | | **95% CI Lower** | | **95% CI Upper** | |  |
| --- | --- | --- | --- | --- | --- | --- | --- | --- | --- | --- | --- | --- | --- | --- | --- | --- | --- |
| **Pre-campaign vs Campaign** | | 0.09 | | 0.97 | | 15.0 | | 0.10 | | 0.99 | | 0.06 | | -1.17 | | 1.29 | |
| **Pre-campaign vs Post-campaign** | | -3.43 | | 0.97 | | 15.0 | | -3.53 | | **0.01** | | -2.04 | | -3.50 | | -0.57 | |
| **Campaign vs Post-campaign** | | -3.52 | | 0.97 | | 15.0 | | -3.62 | | **0.01** | | -2.09 | | -3.57 | | -0.62 | |

**Interpretation:** no assumption violated. ANOVA found a difference in churn rate across periods (p=0.003, ƞ**^2^**=0.532). Post hoc tests comparisons further **confirmed that difference in loss rate between Campaign vs Pre-campaign (p_tukey_=0.008, Cohen’s d-=-2.035, very large negative effect size) and Post-campaign vs Pre-campaign (p_tukey_ =0.007, Cohen’s d=-2.093, very large negative effect size) periods**.

1. **User base retention: proxy: avg vs period**

| ANOVA - User base retention :proxy :avg | | | | | |  |
| --- | --- | --- | --- | --- | --- | --- |
|  | **Sum of Squares** | **df** | **Mean Square** | **F** | **P** | **Ƞ^2^** |
| **Period** | 48.3 | 2 | 24.17 | 8.53 | **0.003** | **0.532** |
| **Residuals** | 42.5 | 15 | 2.84 |  |  |  |

**Assumption checks**

| Homogeneity of variances test (Levene's) | | | |
| --- | --- | --- | --- |
| **F** | **df1** | **df2** | **p** |
| 2.81 | 2 | 15 | **0.092** |

| Normality test (Shapiro-Wilk) | |
| --- | --- |
| **Statistic** | **p** |
| 0.972 | **0.842** |

**Post hoc analysis**

| **Period** | **Mean Difference** | | **SE** | | **df** | | **t** | | **p_tukey_** | | **Cohen's d** | | **95% CI Lower** | | **95% CI Upper** | |  |
| --- | --- | --- | --- | --- | --- | --- | --- | --- | --- | --- | --- | --- | --- | --- | --- | --- | --- |
| **Pre-campaign vs Campaign** | | -0.09 | | 0.97 | | 15.0 | | -0.10 | | 0.99 | | -0.06 | | -1.29 | | 1.17 | |
| **Pre-campaign vs Post-campaign** | | 3.43 | | 0.97 | | 15.0 | | 3.53 | | **0.01** | | 2.04 | | 0.57 | | 3.50 | |
| **Campaign vs Post-campaign** | | 3.52 | | 0.97 | | 15.0 | | 3.63 | | **0.01** | | 2.09 | | 0.62 | | 3.57 | |

**Interpretation:** no assumption violated. ANOVA found a difference in loss rate across periods (p=0.003, ƞ**^2^**=0.532). Post hoc tests comparisons further **confirmed that difference in user base retention between rate between Campaign vs Pre-campaign (p_tukey_=0.008, Cohen’s d-=-2.035, very large negative effect size) and Post-campaign vs Pre-campaign (p_tukey_ =0.007, Cohen’s d=-2.093, very large negative effect size) periods**.
