# Supplementary material for: Implementation of a marketing plan for the dissemination of the WHO SkinNTDs app in Cameroon
Source: PLoS One. 2025 Sep 25;20(9):e0333295. doi: 10.1371/journal.pone.0333295 (PMC12463274; doi:10.1371/journal.pone.0333295)
Supplement: Attachment — Submitted filename: Response to Reviewers.docx [file pone.0333295.s008.docx]

Yaounde, August 15, 2025

**Henri Claude MOUNGUI**

Yaounde – Cameroon

Tel: +237 672192283 - E-mail: henrimoungui@yahoo.fr

**To the Academic Editor**

**of PLOS ONE journal**

**Subject: Rebuttal letter - Manuscript [PONE-D-25-31736]**

Dear Editor,

On behalf of our research team, I am pleased to submit the revised version of our manuscript entitled *“****Implementation of a marketing plan for the dissemination of the WHO SkinNTDs app in Cameroon****”*. We sincerely appreciate the constructive feedback from the reviewers and have thoroughly addressed all comments. Below is a summary of key revisions:

**Major improvements**

1. **Enhanced methodological rigor**
   - Added **interrupted time-series analyses** (ARIMA, GAMM, Poisson GLMM) to address secular trends and strengthen causal inference (Reviewers 1.3, 2.5).
   - Clarified statistical procedures (Tukey’s HSD, DSCF tests) and model diagnostics (AIC/BIC, Ljung-Box) for multiple comparisons (Reviewers 1.7, 1.8, 2.7).
2. **Strengthened theoretical framework**
   - Anchored the dissemination strategy in **Diffusion of Innovations (DOI) Theory** and the **Technology Acceptance Model (TAM)** (Reviewer 2.1).
3. **Expanded transparency**
   - Detailed limitations of Google Play data (**Android vs. iOS market share**, sideloading) and added cost-effectiveness as future work (Reviewers 1.4, 1.6).
   - Provided WhatsApp engagement metrics (message frequency, media exchanges) and A/B testing protocols (Reviewers 1.9, 2.9).
4. **Clarified campaign design**
   - Defined **SMART objectives**, sampling strategy (convenience sampling), and fidelity limitations (Reviewers 2.2, 2.4, 2.10).
   - Added supplementary materials (video outlines, refined content) (Reviewer 2.6).

**Minor revisions**

- Corrected grammar, citations, date formats, and effect-size reporting (Reviewers 1.10, 2.8).
- Expanded discussion of sustainability, observer bias, and formative assessment gaps.

All changes are tracked in the manuscript and itemized in the "Response to Reviewers" document. Supplementary files (statistical code, videos, appendices) are also updated.

We believe these revisions significantly strengthen the manuscript and thank you for your consideration.

Sincerely,

**Henri Moungui**

Below is a table with detailed responses to each reviewer’s comment.

**Reviewer 1**

| **Comment #** | **Comment Summary** | **Suggested Response** |
| --- | --- | --- |
| 1 | Short Follow-Up Period :  The follow-up period post-intervention is limited to six months. This is insufficient to assess the long-term sustainability of the intervention, especially since most gains appear concentrated in the campaign phase. A longer follow-up or at least a more detailed discussion of sustainability challenges is warranted. | We have acknowledged the limitation and expanded discussion:  We agree that the 6-month follow-up period limits assessment of long-term sustainability. While this timeframe may suffice to evaluate adoption during a time-bound mass drug administration (MDA) campaign for NTDs—where short-term engagement is critical—it is insufficient to gauge sustained use beyond the intervention phase. Integration into national programs (e.g., recurring MDA campaigns) could improve long-term viability. Future studies should track retention over extended periods (≥12 months) to assess institutionalization. |
| 2 | Population Selection Bias and Observer Effect:  The manuscript acknowledges the possibility that some users may not be FHWs, but this issue is underexplored. Additionally, since many participants were actively engaged through WhatsApp and in-person presentations, there is a high risk of observer effect (Hawthorne effect), where participants modify behavior due to awareness of being observed, which is not discussed sufficiently. | Thank you for this suggestion. We have added the following in Limitations:  We targeted FHWs but included third-year nursing students as a proxy subpopulation, as trainings occurred in both clinical (health facilities/program meetings) and academic (nursing schools) settings. A companion study (under review) found no significant difference in the app quality mean scores between professionals and students, supporting the validity of this proxy approach.  Observer bias (Hawthorne effect), may have occurred due to active engagement via WhatsApp and in-person sessions, potentially inflating short-term app usage. While post-campaign attrition suggests this effect was temporary, we cannot rule out behavioral modifications (e.g., increased self-reporting). Future studies should minimize observer presence and verify user roles through in-app registration. |
| 3 | Lack of a Control Group:  Without a control group (e.g., a comparable region where no dissemination activities were conducted), it is difficult to attribute observed increases in app downloads or retention to the intervention rather than external factors or secular trends. A stronger justification of the quasi-experimental design, or acknowledgment of this limitation, is necessary. | We acknowledge secular trends (e.g., WHO advocacy) could influence results. For example, WHO NTDs team disseminated the existence of the app through its website and sending emails to our international networks, over 1000 people in their data base. The team members also talked about the app during the meetings they organized or attended.  However, pre-campaign baselines showed minimal activity. Resource limitations precluded control sites.  We have also performed additional interrupted time series analyses to investigate the effect of the campaign in Cameroon and compared Cameroon’s data to other countries.  This is now reported in Methods, Results and Discussions sections.  Thank you. |
| 4 | Exclusive Reliance on Google Play Data:  All quantitative app engagement metrics are derived exclusively from Google Play Console, ignoring iOS users and informal app-sharing mechanisms common in Low- and Middle-Income Countries settings (e.g., Xender). This likely results in underreporting of actual app adoption. The authors should clarify the proportion of Android vs. iOS devices in the target population and better justify the reliance on Android data alone. | We thank the reviewer for this comment. We have now added in Limitations:  “Google Play Console metrics—such as Install Events, Install Base, DAU/MAU, Device Acquisition, Uninstall Events, and User Acquisition—capture Android installs regardless of distribution channel, including sideloaded installations via Xender. However, none of these metrics track iOS users, as installations on Apple devices fall completely outside the Google Play ecosystem. Moreover, metrics tied to the Play Store listing (Store Listing Visitors, Store Listing Acquisitions, Device Store Listing Impressions) cannot capture sideload installations or iOS installs. We argue that this limitation had minimal impact due to Android’s superior market penetration, except on store conversion rates.”  We also added in Methods (Data collection and analysis), the proportion of Android vs. iOS devices in Cameroon: “Android and iOS are the dominant mobile operating systems in Cameroon, with Android holding 79–84% of the market share and iOS representing 14–20% during the pre- and post-campaign period. Given Android’s dominance, we focused solely on Android for this study.” |
| 5 | No Measure of Clinical Impact :  The study focuses entirely on process outcomes (downloads, installs, retention) without assessing any impact on clinical practice or health outcomes (e.g., improved diagnosis or reporting of sNTDs). The absence of even a basic qualitative assessment of changes in practice is a significant limitation that must be more thoroughly acknowledged. | Tracking changes in practice among FHWs who adopted the app was beyond the objectives of this study. Future studies on the app should investigate the app’s contribution to clinical effectiveness.  We have mentioned this as the Future directions section in Discussions. |
| 6 | No Cost-Effectiveness Analysis:  There is no data on cost per download, cost per retained user, or resource expenditure relative to outcomes. Given the relevance of cost-effectiveness for decision-making in LMIC health systems, this represents a missed opportunity. At minimum, the authors should discuss this limitation more openly. While the app is freely available, the implementation of the marketing strategy incurred costs (staff salaries, transportation, in-person events, communication materials). The paper does not account for these costs relative to key outcomes (e.g., cost per additional active user, cost per retained user). Without this, policymakers in similar settings cannot assess the affordability or scalability of the approach. I recommend either a basic cost analysis or a transparent acknowledgment of this omission in the limitations. | We acknowledge the importance of cost-effectiveness data for policy decisions. A formal cost-effectiveness analysis was precluded by the app's early-stage evaluation (WHO stages 1-2 of digital intervention maturity). As this was a proof-of-concept dissemination study, we prioritized adoption metrics over economic evaluation. Future scale-up studies should incorporate metrics such as cost-per-install, or cost-per-case detected, particularly when transitioning to WHO stages 5-6 (Scale-up/Integration/sustainability).  This is now mentioned in limitations. |
| 7 | Use of Basic Statistical Tools:  The choice of Microsoft Excel and Jamovi for statistical analysis raises concerns about analytical robustness. While acceptable for basic ANOVA and non-parametric tests, the use of more sophisticated statistical packages (e.g., R or STATA) could enable richer analyses such as time-series modeling or interrupted time series designs, which seem more appropriate for before-after quasi-experimental data. | Thank you for the suggestion. We have now clarified in Methods and reported the findings in the Results section.  We conducted three interrupted time series (ITS) analyses to evaluate the impact of the SkinNTDs app dissemination campaign in Cameroon. First, only for Cameroon, an auto regressive integrated moving average (ARIMA) error structure (ARIMA(2,0,0)) modeling both level and slope changes during and post-campaign while accounting for autocorrelation. Second, a generalized additive mixed model (GAMM) compared Cameroon to Nigeria, Ghana, and Ethiopia, to flexibly model nonlinear pre/post-intervention trends in Install base while controlling for country-level heterogeneity. Third, in another cross-country comparison (Cameroon vs Nigeria, Ghana, Ethiopia), a Poisson generalized linear mixed-effects model (GLMM) assessed daily active devices (DADs) with country random effects and interaction terms for intervention effects. Being all endemic to NTDs, the three countries were selected because they were top five African countries with highest install base average values during the 18-months period. During that period, these countries also had similar mobile operating system market share with Cameroon.  Model selection used Akaike/Bayesian Information Criteria (AIC/BIC). Residual diagnostics included Ljung-Box tests, ACF plots, and Q-Q normality checks. These analyses were performed in R 4.5.0 (α = 0.05) and full descriptions of ITS procedures including model specifications are available in supporting information file S3 Appendix. |
| 8 | Lack of Clarity on p-Value Correction :  The manuscript does not sufficiently describe whether p-value corrections for multiple testing were applied, especially given multiple outcome metrics. While the use of Dwass-Steel-Critchlow-Fligner tests is appropriate for pairwise comparisons, there is no clarity on overall correction for Type I error across multiple comparisons, which is critical in studies with numerous endpoints. | Significant omnibus results were followed by pairwise comparisons: Tukey’s Honestly Significant Difference (HSD) test for ANOVA when assumptions were met, and the Dwass–Steel–Critchlow–Fligner (DSCF) test for Kruskal–Wallis outcomes when assumptions were not met.  This is now elaborated in Methods section (Inferential statistics sub-section). |
| 9 | Inadequate Insight on WhatsApp Group Dynamics:  The paper rightly identifies WhatsApp as a key engagement tool, yet offers limited analysis of group activity (e.g., participation rates, message frequency, engagement levels over time). Some quantitative or qualitative reporting on WhatsApp engagement would substantially strengthen the insights about sustained app use. | During the campaign, 227 messages were exchanged in the WhatsApp group. Message frequency peaked on Mondays (n = 51), with the highest activity occurring in September 2024 (n = 113) and July 2024 (n = 79). Most messages were sent during morning (8:00–10:59 AM) and evening (5:00–8:59 PM) periods. The primary co-author was the most active participant, and the Google Play Store link to the app was shared six times in the group (three times each in May and July 2024). Additionally, 98 media files—including instructional videos, PDFs, screenshots, and field photos—were exchanged. Beyond the group, the primary author promoted the app individually, sharing the Play Store link approximately 50 times in private chats.  This is now clarified in Results section. Thank you. |
| 10 | 1) Reference [3] appears in the reference list but is not cited in the main text. This should be corrected. 2) Statistical significance is sometimes highlighted without consistent emphasis on effect sizes or practical significance, which would be more informative to readers.  3) Edit grammar (e.g., "less favorable dynamics were evident" → "dynamics were less favorable"). Some sentences would benefit from minor grammatical editing for conciseness and clarity. | 1)Reference [3] already corrected.  2) We reported effect sizes to show practical importance through Cohen’s d for t-tests (with 95% confidence intervals), or Eta-squared (η²) for ANOVA.  3) Checked sentences for grammatical editing for conciseness and clarity. |

**Reviewer 2**

| **Comment #** | **Comment Summary** | **Suggested AI Response** |
| --- | --- | --- |
| **1** | The description of the marketing plan outlines various dissemination channels (WhatsApp, printed posters, face‐to‐face training, emails) but does not anchor these activities in a recognized health behaviour or communication theory (e.g., Health Belief Model, Diffusion of Innovations). Explicitly identify and describe a theoretical framework that guided message development and channel selection. This will strengthen the rationale for why certain campaign components were expected to influence FHW knowledge, attitudes, or practices. | We have added a subsection “Theoretical framework” under Methods section:  “The study’s dissemination strategy drew on two behavioral frameworks: the Diffusion of Innovations (DOI) Theory, and the Technology Acceptance Model (TAM). DOI guided the campaign’s structure, including phased rollout (launch, engagement, embedment) and channel selection (e.g., WhatsApp for peer networks, in-person training for observability), aligning with its constructs of relative advantage and social system diffusion. TAM informed messaging to highlight perceived usefulness (e.g., diagnostic accuracy) and ease of use (e.g., offline functionality, ease of download/installation), targeting FHWs’ behavioral intent. A/B testing of materials (e.g., video formats) further optimized compatibility and perceived value, reinforcing theoretical grounding.”  Thank you |
| **2** | Although a “marketing plan” is mentioned, there is no clear statement of SMART (Specific, Measurable, Achievable, Relevant, Time‐bound) objectives (e.g., “Increase app downloads among FHWs in Region X by 25% within three months”).Include a table listing precise campaign objectives (e.g., target reach, frequency of message exposure, engagement metrics) and corresponding process and outcome indicators (e.g., number of posters displayed, number of WhatsApp broadcasts sent, click‐through rates). | We have clarified overall campaign objectives in the draft marketing plan and in the Methods section. we have also discussed how far those objectives were reached.  1. Increase awareness: Reach ≥80% of target FHWs in North/Far North Cameroon via WhatsApp, emails, or in-person sessions within 6 months: 100% of health districts in both regions were contacted but the percentage of FHWs reached was not measured.  2. Drive downloads: Achieve a 60% conversion rate from store visits to downloads (achieved: 64.1%).  3. Sustain engagement: Maintain ≥80% user retention 3 months post-installation (achieved: 88.7% during campaign).  4. Training coverage: Conduct ≥20 in-person sessions (achieved: 12 sessions + 18 facility visits). |
| **3** | The methods lack any mention of formative assessment (e.g., focus groups, key‐informant interviews) or pretesting of messages/materials with the target audience before full rollout. Describe any formative work undertaken to tailor content (language, visuals) to the local context and document how A/B testing informed final message versions. If no formative research was done, discuss this as a limitation. | We have revised the manuscript to include a description of the A/B testing process used to refine the main dissemination video in the Methods section, the corresponding feedback and recommendations from participants in the Results section, and reflections on its implications in the Discussion. Although this process allowed us to tailor visual elements, pacing, and content clarity to the local context, we did not conduct a systematic formative assessment (e.g., focus groups or key informant interviews) for other dissemination materials. This limitation is now explicitly acknowledged in the Discussion section. |
| **4** | There is no information on how the fidelity (i.e., whether activities were delivered as planned) or actual exposure of participants to each channel was monitored. Detail procedures for tracking campaign delivery (e.g., logs of WhatsApp broadcast times, attendance sheets for workshops, distribution records for posters) and how exposure data were linked to individual app‐usage metrics. | Now in limitations: Attendance sheets for workshops were maintained. However, information on how the fidelity (i.e., whether activities were delivered as planned) or actual exposure of participants to each channel was not monitored. Exposure data were not linked to individual app‐usage metrics. This should be tracked into future studies.  Thank you. |
| **5** | The before–after design without a contemporaneous control group is vulnerable to secular trends (e.g., global publicity, other local NTD initiatives) that might influence app uptake. Acknowledge and discuss potential confounders (e.g., simultaneous WHO or NGO activities), and consider whether any comparison districts without targeted marketing could have been used as controls. | We acknowledge secular trends (e.g., WHO advocacy) could influence results. For example, WHO NTDs team disseminated the existence of the app through its website and sending emails to our international networks, over 1000 people in their data base. The team members also talked about the app during the meetings they organized or attended.  However, pre-campaign baselines showed minimal activity. Resource limitations precluded control sites.  We have also performed additional interrupted time series analyses to investigate the effect of the campaign in Cameroon and compared Cameroon’s data to other countries.  This is now reported in Methods, Results and Discussions sections.  Thank you. |
| **6** | The methods state that “videos and presentations” were used, but offer no specifics on content length, format, language, or whether materials were adapted for literacy levels. Provide sample outlines or screenshots of videos/slides (e.g., durations, main messages), and describe how materials were localized (translations, pictograms) for FHW audiences. | The file of Video A is available in supplementary materials. The narrative content of both Video A and B is also available in a separate file. Materials were developed in French/English versions and adapted to the audience throughout the campaign.  This is now clarified in a new “Dissemination materials refinement process” sub-section in Methods. Thank you. |
| **7** | While the use of ANOVA and non‐parametric tests is described, there is no mention of how missing data (e.g., uninstalls, silent non-users) were handled or whether any adjustments were made for multiple comparisons across three periods.Specify data-cleaning rules, handling of outliers, and correction methods (e.g., Bonferroni) if multiple pairwise contrasts were conducted. Consider presenting an a priori power calculation for detecting meaningful changes in download or retention rates. | We appreciate the reviewer’s suggestion. As noted in the revised Methods section, the Google Play Console data are provided in an anonymized, pre-aggregated format, and therefore no raw-level cleaning or outlier removal was possible or necessary. Because the dataset contains only aggregated metrics (e.g., period-level download, installation, and retention rates), there were no individual-level missing data to address. All uninstallations and silent non-users were already reflected in the aggregated metrics as defined by Google Play Console. For multiple comparisons, we used Tukey’s HSD for ANOVA when assumptions were met and the Dwass–Steel–Critchlow–Fligner procedure for nonparametric outcomes; both control the family-wise error rate within each outcome’s pairwise comparisons. We acknowledge that an a priori power calculation could not be conducted due to the absence of prior estimates for expected effect sizes and variability in these specific Google Play Console metrics; however, we have now noted this limitation in the Discussion. |
| **8** | The six‐month pre-, during-, and post-campaign windows are clear, but dates in the text (e.g., “October 2024–March 2025”) should be formatted consistently (e.g., “1 October 2024 to 31 March 2025”). | Corrected. Thank you. |
| **9** | Provide specifics on how A/B tests were designed (randomization process, sample sizes per variant, success criteria), and indicate which version emerged as optimal. | We have updated the Methods section to describe the A/B testing process for the main dissemination video, including participant selection (10 purposefully selected stakeholders from central, regional, and district levels), feedback collection procedures, and the criteria used to assess clarity, length, and suggested improvements. The Results section now summarizes participants’ sentiments and key recommendations, and the Discussion reflects on how these informed refinements to the final video version. While feedback allowed us to enhance visual clarity, pacing, and contextual relevance, we did not implement randomization, use balanced sample sizes per variant, or formally define quantitative success criteria. Only one variant (Video B) incorporated the recommended changes and was retained for rollout. The absence of a systematic formative assessment and full experimental A/B design for other dissemination materials is now noted as a limitation. |
| **10** | Explain how frontline health workers were identified and invited to participate in the campaign—was it purposive, convenience, or exhaustive sampling within target districts? | FHWs were identified and invited to participate in the campaign in convenience sampling within target districts. The reason behind was to adapt with operational feasibility and to stick with the idea of integration into the health system. Thus, the research team was constantly looking for opportunities when programs in the ministry of health organized training sessions or meeting with FHWs.  This is now clarified in Study design section. Thank you. |
