# Supplementary material for: Implementation of a marketing plan for the dissemination of the WHO SkinNTDs app in Cameroon
Source: PLoS One. 2025 Sep 25;20(9):e0333295. doi: 10.1371/journal.pone.0333295 (PMC12463274; doi:10.1371/journal.pone.0333295)
Supplement: Attachment — Submitted filename: Response_to_Reviewers_auresp_2.docx [file pone.0333295.s009.docx]

Yaounde, September 2, 2025

**Henri Claude MOUNGUI**

Yaounde – Cameroon

Tel: +237 672192283 - E-mail: henrimoungui@yahoo.fr

**To the Academic Editor**

**of PLOS ONE journal**

**Subject: Rebuttal letter - Manuscript [PONE-D-25-31736]**

Dear Editor,

On behalf of our research team, I am pleased to submit the revised version of our manuscript entitled *“****Implementation of a marketing plan for the dissemination of the WHO SkinNTDs app in Cameroon****”*. Thank you for the opportunity to revise our manuscript and for the constructive feedback provided by the reviewers. We have carefully considered all comments and have made significant revisions to the manuscript to address them. Our point-by-point responses are detailed below.

**Response to Reviewer Comments:**

**Reviewer 1:**

**Comment 1:** *"You report using Tukey’s HSD (after ANOVA) and DSCF (after Kruskal–Wallis), which controls Type I error within each outcome’s pairwise comparisons. Please clarify whether you also applied multiplicity control across outcomes (e.g., growth, churn, retention, loss, device conversion). Add one explicit sentence in Methods (and mirror it in Limitations, if applicable) stating whether across-outcome correction was or was not performed."*

**Response:** We thank the reviewer for this crucial methodological point. We have now explicitly addressed this in the manuscript as follows:

1. **Methods Section (Inferential statistics):** We have added the sentence: *"While Tukey’s HSD and DSCF tests controlled the Type I error rate for pairwise comparisons within each outcome, no statistical correction for multiple testing (e.g., Bonferroni, Holm, or FDR) was applied across the six primary outcomes."*
2. **Limitations Section:** We have added a statement acknowledging this limitation: *"We did not adjust for multiplicity across the six different outcome metrics, which increases the risk of Type I errors across the study's findings. However, the large effect sizes (e.g., Cohen’s d > |1.5|) and consistent pattern of results for key metrics strengthen our confidence in their validity."*

**Comment 2:** *"Optional: a brief sensitivity note, e.g., Holm or FDR across omnibus p-values, would be welcome but is not required."*

**Response:** We thank the reviewer for this helpful suggestion. We performed the requested sensitivity analysis. A Bonferroni correction (adjusted α = 0.0083) confirmed that the significant period effects for **growth rate (p = 0.007), churn rate (p = 0.003), and user retention rate (p = 0.003)** remained robust. The effect for loss rate (p = 0.011) did not survive this conservative correction. We have added a brief note on this in the **Results** section.

All changes have been tracked in the manuscript file. We believe these revisions have significantly strengthened the paper's methodological rigor and transparency.

As per the editorial request, we have thoroughly reviewed our reference list to ensure its completeness and correctness. We confirm that:

1. All references are current, relevant, and accurately cited. We added one reference on Cameroon’s background data (health system and NTDs management structure), and one on statistical correction for multiple testing.
2. We have verified that none of the cited papers have been retracted. Therefore, no additions or rationales regarding retractions were required.
3. All in-text citations have a corresponding complete entry in the reference list.

Thank you again for your time and consideration.

Sincerely,

**Henri Moungui**
